# President’s Address

**Published:** 1903-10-15

**Authors:** E. A. Honey

**Affiliations:** Kalamazoo


### PRESIDENT'S ADDRESS.

E. A. HONEY, D.D.S., KALAMAZOO.

*To the members of the Michigan Dental Association:* I regret very much that the committee having in charge the revision of the constitution should not have provided a committee to furnish the President's address, at least for the present incumbent. If little new or original should be contained herein you will kindly lay the blame at the door of this committee.

Speaking of the work of committees, I believe some of us scarcely realize the importance of the work they accomplish in these societies. I would suggest that the number of these committees be increased, at least one on membership might profitably be added. We meet now, as our program states, to participate in the forty-seventh annual convention of the association, and yet our records show we have added to our membership during these forty-seven years something less than four members per year as a net increase. Polk's *Dental Register* issued last year contains the names of one thousand and twenty dentists residing in the State of Michigan. We will suppose for the time being that one-half of these men are eligible to membership in this Society, with our present membership of 170, it will leave a balance of over 300, or a large field from which to secure new members. I would suggest the establishing of a membership committee, fairly generous in number and selected from different parts of the State, whose duty it should be to see that new men are being brought into the Society in generous numbers each year. One of the greatest favors we can confer on young graduates is to assist in establishing in them the habit of attendance at these gatherings.

I wish to thank the Board of Trustees and the local committee for the work they have done in preparing the program for what promises to be one of the best meetings we have ever had. Our new constitution places the work of preparing the program in the care of the Program Committee and the Clinic Trustee. How well they have performed this duty we shall know when this meeting is over.

The duties of these two trustees, as outlined in the constitution, would seem to be an equitable division of the work expected of them, and perhaps it is; but if so, the number should be increased so far as the clinic trustee is concerned at least, as the work found necessary in looking after material for clinics and taking charge of the exhibits with the manifold details that go with such work has proved too much for one person. It would seem that this work might be divided, giving one member charge of the clinics, and placing the exhibits in the hands of an additional member, elected or appointed for that purpose. When we state that from 150 to 200 personal letters have been written in order to secure the number of clinicians on our program, it will scarcely seem unreasonable that the trustee having this work in charge should ask to be relieved of some of the other work.

Another matter which must be decided at this meeting is whether or not we shall continue the Tri-State meetings which have been held at regular intervals since 1895, at which time the Michigan dentists were hosts. Few I think will question the advisability of continuing these meetings who have participated in them in the past, but as you all know in 1904 will be held in St. Louis in connection with the Universal Exposition an International Dental Congress similar in scope to the one held in Chicago during the World's

Fair. It is now being organized by a committee appointed by the National Dental Association. Dr. H. J. Burkhart, of Batavia, N. Y., is the chairman of this committee. From a letter received from him a short time ago I quote the following: "The Congress will be international in its character. The International Federation accepted our invitation last year, and we have just received a cable that the British Dental Association had also accepted. We propose to conduct the Congress in a broad-minded and liberal manner so that every one can take part. The details have not yet been worked out, but we expect soon to be in good running order. I should be very glad to have your society assist us in any way possible, and also to receive suggestions as the proper persons to put on the various committees. We should of course be pleased to receive any financial aid your Society can give us."

Now, inasmuch as many of us throughout the State expect to attend this dental congress at St. Louis, it has been thought by some that it would be advisable to postpone the Tri-State meeting for one year. Definite action on this matter should be taken at this time and the Indiana and Ohio Societies notified accordingly.

A year ago the President of this Society in his annual address called attention to the importance of appointing a body of practicing dentists either by the Governor or the Mayor of our cities whose duty it would be to examine the teeth of the children in our public schools. In no way could we be of greater service to the community at large than to be instrumental in bringing about this result.

The proper care of the teeth of children, in especially the lower grades, will do a great deal to avert diseases resulting from improper mastication and will prevent hundreds of cases of facial deformities brought about by the premature extraction of the temporary teeth, to say nothing of the good accomplished by saving the first permanent molar. As this Society is the only organized body of dentists embracing the entire State, it would seem to be its duty to make the first move.

Hundreds of little patients come to us every year for whom we can do little or nothing. They are suffering from a nervous irritability due partially to gastric disturbances which are the result of imperfect mastication made necessary by the decay or loss of the temporary teeth or first permanent molar or both. If the teeth of these little people could be examined while in the kindergarten, a report issued for each showing the exact conditions and placing same in the hands of the parents, it would result in inestimable good.

It is pleasing to note the progress that has been made along some of the special branches in our profession even during the past year or two years.

Orthodontia is receiving more and better attention than ever before, yet hundreds of almost pitiful cases of irregularities and facial deformities are being grossly neglected, mainly due to the lack of sufficient intelligence along this line of work to give them the attention they should receive. The men devoting their time to this work should receive the hearty support of the profession, and more dentists should be urged to go into this service.

The enthusiasm aroused in porcelain work during the past year is almost marvelous. Scores of men throughout the country are taking it up and many of them are meeting with gratifying results. The pioneers in this work grow more confident every year of its merit and permanence, and their records show very clearly that it is no longer an experiment.

We have among our members those who have gained more than a local reputation in oral surgery. Special study along this particular branch has enabled these men to successfully treat many cases that baffle the skill of the general practitioner, and it should be with a sense of pride and not of apology that we refer such cases to them.

The State Board of Dental Examiners has been doing good work, and gives the following report covering the past eighteen months. During that time fifty men have taken the examination, of these forty were graduates, three had finished three years in college, one had not graduated, but had completed two years in college, and six had never been to college—of these forty-three passed. Of the seven that failed, four were graduates, one had attended college, and two had never attended. Eighty-five men have registered as graduates from Michigan colleges. This year 126 have graduated from Michigan colleges, of these sixty-five have registered since their graduation in June.

The Secretary of the State Board complains of the lack of support from local dentists in prosecuting illegal practitioners. I will quote his exact words on this point:

"The Board has endeavored to adjust all cases brought to its notice. It meets with a little support from the men making the complaints. They invariably ask that we keep their names secret, and this defeats any means used to secure conviction, as prosecuting attorneys will not act unless they have local support. In all cases an affidavit has been sent that the men complained of were not registered. In most cases the complaining dentist let it drop, failing to recognize the fact that if the conviction is secured that not only have they attained the end sought, but the member of the Board if summoned should at least have his expenses paid." It is much to be regretted that any of us should attempt to shirk responsibilities of this kind. It should be the urgent duty of every member of this Society to co-operate in every way possible with the State Board and especially in this branch of work, in fact, the two bodies should be very closely allied. The inter-dependence of these two bodies has been so recognized in the State of Indiana that their dental law provides for the appointing by the State Society of three of the five members of the State Board of Dental Examiners; one appointment being made by the State Board of Health, and one by the State Executive. This furnishes us with a good suggestion upon which to work to amend our present law in this State. The profession and not the State Executive should be responsible to the people for the appointments made on this Board. It would relieve the Governor of much embarrassment that has sometimes been brought about in the past when appointments have been secured solely by virtue of the political influence brought to bear in behalf of the member receiving such appointment.

These remarks are in no way intended as a criticism on the present members of the Board, nor to open up a gap between them and this association; but merely the reiteration of a principle for which the Society has always stood and for which I hope it will continue to stand until such time as we are enabled to wield a sufficient influence to bring about such legislation as will place the responsibility for these appointments where they rightfully belong.

